# Lower limb biomechanical differences between forehand and backhand forward lunges in amateur female badminton players

**DOI:** 10.3389/fbioe.2025.1558918

**Published:** 2025-02-21

**Authors:** Zhonghao Xie, Jing Pan, Xingyu Wu, Huiting Liang, Bosi Chen, Dongping Tan, Meng Wu, Zhiguan Huang

**Affiliations:** ^1^ Guaduate School, Guangzhou Sport University, Guangzhou, China; ^2^ School of Competitive Sports, Guangdong Vocational Institute of Sport, Guangzhou, China; ^3^ Guangzhou Institute of Sports Science, Guangzhou, China; ^4^ School of Sports and Health, Guangzhou Sport University, Guangzhou, China

**Keywords:** badminton, lunge, female, lower limbs, biomechanics

## Abstract

**Background:**

Forehand and backhand forward lunges are frequently performed in badminton, placing significant demands on the lower limbs. The purpose of this study was to examine the differences in lower limb biomechanics between these two lunge types in female amateur players.

**Methods:**

This study involved 17 female amateur badminton players performing forehand and backhand forward lunges. Lower limb kinematics and dynamics were recorded using an eight-camera Vicon motion capture system and two AMTI force plates. Variables such as joint angle, range of motion, stiffness, and ground reaction forces measured during the stance phase were analyzed using paired t-tests. To account for the one-dimensional nature of joint angles, moments, and ground reaction forces, the analysis was performed using paired sample t-tests in Statistical Parametric Mapping 1D.

**Results:**

The forehand lunge exhibited a smaller hip flexion angle, greater hip internal rotation angle, and increased hip stiffness compared to the backhand lunge. The backhand lunge, in contrast, demonstrated a higher ankle varus angle and greater transverse plane hip range of motion. SPM1D analysis revealed significant differences in both the early (0%–10%) and late (80%–100%) phases of the stance phase. In the early phase, the backhand lunge showed a larger internal rotation moment at the hip, an external rotation moment at the knee, and a smaller knee extension moment. In the late phase, the forehand lunge revealed greater internal rotation moments at the hip, external rotation moments at the knee, ankle valgus moments, and smaller knee flexion moments.

**Conclusion:**

The backhand lunge requires greater hip internal rotation than the forehand lunge. Additionally, it is associated with higher ankle varus angles, which may increase the risk of ankle injuries. In contrast, the forehand lunge demonstrates greater hip stiffness, potentially reflecting an adaptation of the lower limb to varying directional demands. These findings emphasize the importance of incorporating targeted ankle and hip training exercises into conditioning programs.

## 1 Introduction

Badminton is one of the most popular sports globally, attracting participants of all ages, genders, and skill levels (Phomsoupha and Laffaye, 2015; Mei et al., 2017). Mastering effective footwork is essential for players to position themselves optimally for shots and swiftly return to the base position in preparation for opponents’ returns (Mei et al., 2017). Among all footwork types, lunges are particularly common, constituting over 15% of all movements (Kuntze et al., 2010; Hong et al., 2014; Mei et al., 2017). Executing lunges requires high muscle activity and substantial core and knee dynamic stability to manage rapid changes in body position (Phomsoupha and Laffaye, 2015; Lam et al., 2020). This demanding footwork might be partially responsible for the high risk of injuries to the knee and ankle joints (Herbaut et al., 2018; Lam et al., 2020). Consequently, conducting in-depth research on lunges is essential to further understand the biomechanical demands and injury prevention strategies associated with these movements.

Lunges in badminton can be categorized into four primary directions (Hong et al., 2014), with forehand and backhand forward lunges being the most critical due to their high frequency in gameplay (Hong et al., 2014; Hu et al., 2015; Lee and Loh, 2019; Valldecabres et al., 2020). Previous studies have demonstrated that forward lunges in different directions exhibit distinct dynamic characteristics (Hong et al., 2014; Nielsen et al., 2020). For instance, Hong et al. (2014) reported that backhand forward lunges generate higher ground reaction forces and plantar pressures in right-handed badminton players compared to forehand lunges, suggesting a higher injury risk during backhand lunges. Conversely, Nielsen et al. (2020) found that backhand lunges are associated with significantly lower hip, knee, and ankle frontal plane moments compared to forehand lunges, potentially indicating a reduced risk of overuse injuries and discomfort. These conflicting findings underscore the need for further research to clarify the biomechanical differences between forehand and backhand forward lunges.

The badminton lunge is a closed-chain movement that involves simultaneous flexion and extension at the hip, knee, and ankle of the dominant limb (Maloney, 2018). This movement can be described within the framework of the stretch-shortening cycle (SSC), which is essential for developing sufficient lower-limb stiffness to store elastic energy and generate force during SSC activities (Komi, 2000; Brazier et al., 2019). Stiffness can be described as the resistance to deformation of an object in response to an applied force (Butler et al., 2003; Pruyn et al., 2014; Brazier et al., 2019). It arises from the interplay of muscles, tendons, ligaments, cartilage, and bones (Butler et al., 2003; Brughelli and Cronin, 2008). Previous badminton research on stiffness has predominantly focused on footwear (Oleson et al., 2005; Luo et al., 2009; Park et al., 2017; Yu et al., 2023), leaving joint stiffness during lunging movements relatively underexplored. A comprehensive understanding of these parameters can provide deeper insights into overall motor function and the mechanics of lunging.

Studies on gender have indicated that female athletes face higher injury risks compared to their male counterparts (Beaulieu et al., 2008; Landry et al., 2009). Specifically, Lam et al. (2018) compared badminton players of different genders and skill levels performing lunge tasks, finding that unskilled female players—defined as those with no formal competition experience and an average of two to 3 years of badminton practice—are more susceptible to lower extremity injuries due to higher impacts during landing. Therefore, further study of unskilled female players is necessary. Although previous research has examined different directions, genders, and skill levels in lunging movements (Hong et al., 2014; Lam et al., 2018; Nielsen et al., 2020), there is a relative lack of studies focusing on the execution of forehand and backhand forward lunges by unskilled female players. Given the biomechanical differences reported in previous studies, a focused investigation of forehand and backhand lunges among amateur female players is warranted. Addressing this gap will offer valuable insights for developing effective training and injury prevention strategies tailored to amateur female players.

Therefore, this study aims to investigate the kinematic and dynamic characteristics of the lower limbs in amateur female badminton players during forehand and backhand forward lunges. The findings will provide a scientific basis for injury prevention and the development of targeted training strategies for this population. We hypothesize that the lower limb three-joint kinematics and dynamics of amateur female badminton players will exhibit different responses to forward lunges in forehand and backhand directions.

## 2 Methods

### 2.1 Participants

This study recruited 17 amateur female badminton players from Guangzhou Sport University. The sample size was determined based on previous biomechanical research studies (Herbaut and Delannoy, 2020). The participants had an average height of 1.65 ± 0.03 m, an average weight of 51.5 ± 4.08 kg, and a body mass index (BMI) of 18.9 ± 1.43 kg/m^2^. Participants were classified as amateur players based on their non-professional status and engagement in recreational badminton activities.

The inclusion criteria were as follows: (1) regular badminton practice (at least 6 hours per week); (2) a minimum of 2 years of playing experience; (3) age between 18 and 24 years; (4) right-handedness; (5) no formal competition experience; and (6) no history of lower limb injury in the past year.

Ethical approval was obtained from the Ethics Committee of Guangzhou Sport University (Approval No. 2024LCLL-106). All participants provided written informed consent prior to the study.

### 2.2 Experimental setup

**FIGURE 1:**
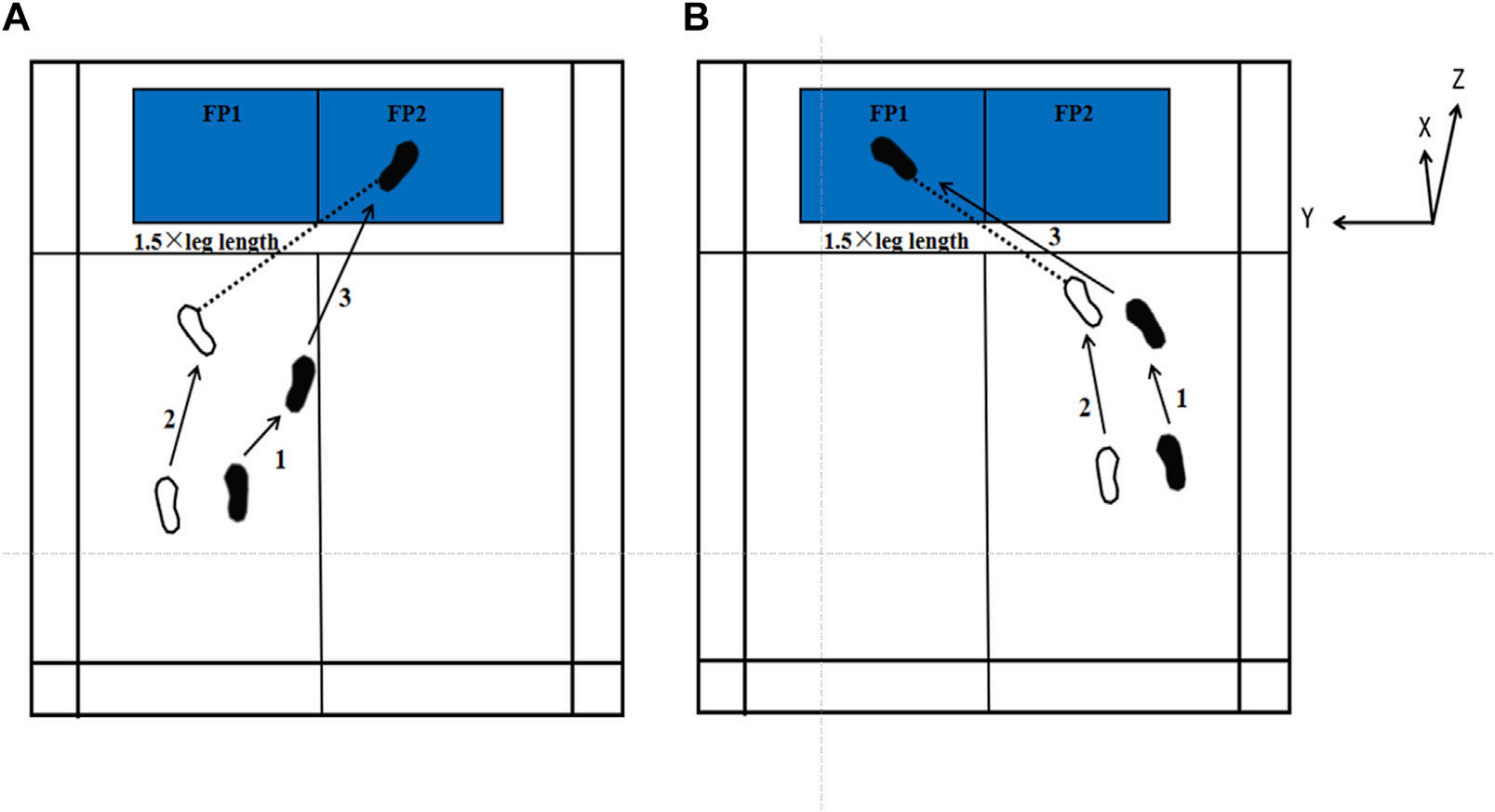
Schematic diagram of forehand and backhand forward lunges. All test tasks were conducted on a simulated badminton court. **(A)** indicates the forehand lunge, and **(B)** represents the backhand lunge. White footprints indicate the position of the left foot, and black footprints indicate the position of the right foot, with numbers indicating the step sequence. FP1 and FP2 represent two different force plates. The dashed line represents 1.5 times the leg length. The X, Y, and Z coordinates are defined according to the force plate.

The experiment was conducted at the Guangdong Sports Equipment Engineering Technology Research Center between October and November 2024. All participants in this experiment performed a three-step lunge technique, incorporating both forehand and backhand lunges (see Figure 1). Kinematic and dynamic data were collected simultaneously. Each data collection session lasted approximately 90 min per participant, including warm-up, equipment setup, and data recording.

#### 2.2.1 Kinematic measurements

**FIGURE 2:**
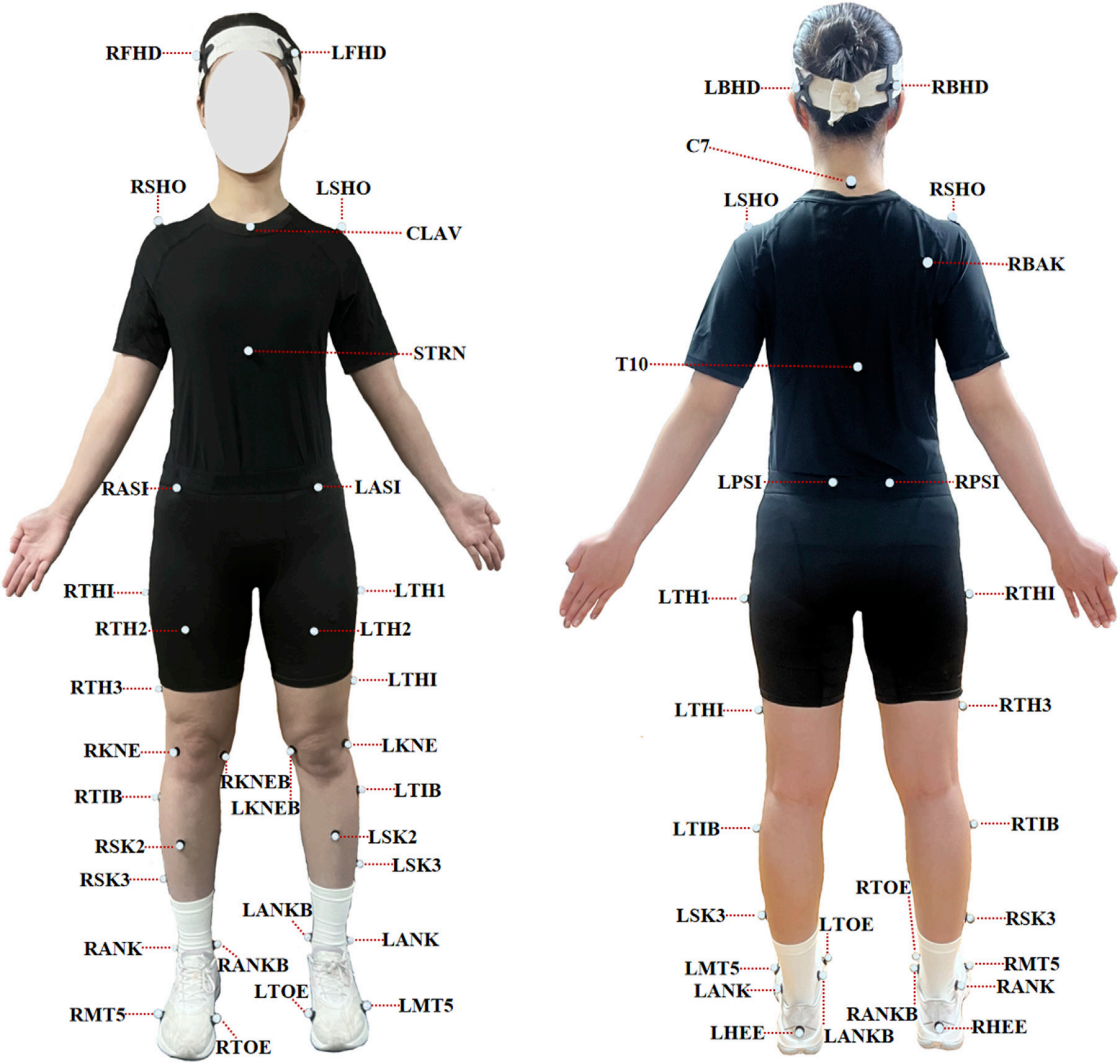
Marker placement.

An eight-camera Vicon motion capture system (Oxford Metrics Ltd., Oxford, UK), sampling at 200 Hz, was utilized to collect raw kinematic data during the badminton lunge. A reflective marker set consisting of 43 markers (diameter: 14 mm), was attached to the participants’ bodies to define joint segments and axes of rotation. To minimize measurement error, all reflective markers were positioned by a single experienced researcher. The marker placement locations included the following anatomical landmarks: the brow bone, occipital bone, acromion, C7 vertebra, center of the right scapula, T10 vertebra, center of the clavicle, lowest point of the sternum, anterior and posterior superior iliac spines, medial and lateral femoral condyles, lateral thigh, lateral calf, medial and lateral malleoli, heel, and the first and fifth metatarsal heads (see Figure 2).

#### 2.2.2 Dynamic measurements

Raw dynamic data were obtained using two AMTI force platforms (Advanced Mechanical Technology Inc., Watertown, MA, United States), operating at a sampling frequency of 1,000 Hz. The force platforms (model OPT-SC) had dimensions of 0.4 m × 0.6 m and were embedded in the floor of a laboratory-simulated badminton court to ensure a level surface for accurate data acquisition. The force plates were zeroed before each trial to ensure data accuracy.

### 2.3 Experimental procedure

#### 2.3.1 Test preparation phase

All participants refrained from engaging in strenuous exercise for 24 h prior to testing. Before the experiment, participants were briefed on the test procedures and safety precautions. To standardize the lunge distance (Kuntze et al., 2010; Nielsen et al., 2020), the starting position for each participant was set at 1.5 times their individual leg length (measured between the anterior superior iliac spine and the lateral malleolus), at an angle of 45° with respect to the x-axis of the force platforms. The designated endpoint was positioned at the center of the force plate. Following this, participants completed a 10-min warm-up, including dynamic stretching and practice lunges to prepare for the formal testing.

#### 2.3.2 Formal testing phase

After receiving the start command, participants held a racket and initiated the lunge from the designated starting position. They were instructed to execute the lunge with maximum effort, simulating competitive conditions as closely as possible. Participants ensured that their racket-holding leg landed on the designated endpoint before quickly returning to the starting position. Each participant performed five valid trials of both forehand and backhand lunges in a randomized order, with the sequence determined using an online randomization tool (www.random.org). A 30-s rest interval was provided between trials to minimize the effects of fatigue. A trial was considered valid if the participant’s front foot landed within the boundaries of the force platform and no noticeable slippage occurred.

### 2.4 Data reduction

Spline interpolation was performed to fill minor missing marker trajectories in the Vicon Nexus 2.15.0 software (Oxford Metrics Ltd., Oxford, UK). All kinematic and dynamic data were subsequently imported into Visual3D software (C-Motion Inc., Rockville, MD, United States) for further processing. Kinematic and dynamic data were filtered using a fourth-order Butterworth filter with cutoff frequencies of 15 and 25 Hz, respectively (Hanzlíková et al., 2016).

The following variables were analyzed: kinematic variables, which included joint angles and joint range of motion (ROM). The three-dimensional kinematics of the joints was calculated using an XYZ Cardan sequence of rotations (X: flexion-extension, Y: abduction-adduction, and Z: internal -external rotation). ROM was defined as the difference between the maximum and minimum joint angles of the hip, knee, and ankle joint during the stance phase. Dynamic variables included ground reaction force (GRF), joint moment, and joint stiffness (Akl et al., 2020; Maloney and Fletcher, 2021). Net joint moment was calculated using the Newton-Euler inverse dynamics approach. Joint stiffness for the ankle, knee, and hip was determined by estimating net joint moments based on inverse dynamics principles and measuring joint angular displacement in the sagittal plane (Maloney and Fletcher, 2021) (Equation 1). A positive value for joint angle and moment denoted hip flexion, adduction, and internal rotation; knee flexion, varus, and internal rotation; ankle dorsiflexion, inversion, and adduction for respective orthogonal planes.

$$K_{\text{joint}}=\frac{∆M}{∆\theta }$$ (1)

where K_joint_ represents joint stiffness, ΔM is the difference between the maximum and minimum joint moments; and Δθ is the difference between the maximum and minimum joint angles.

**FIGURE 3:**
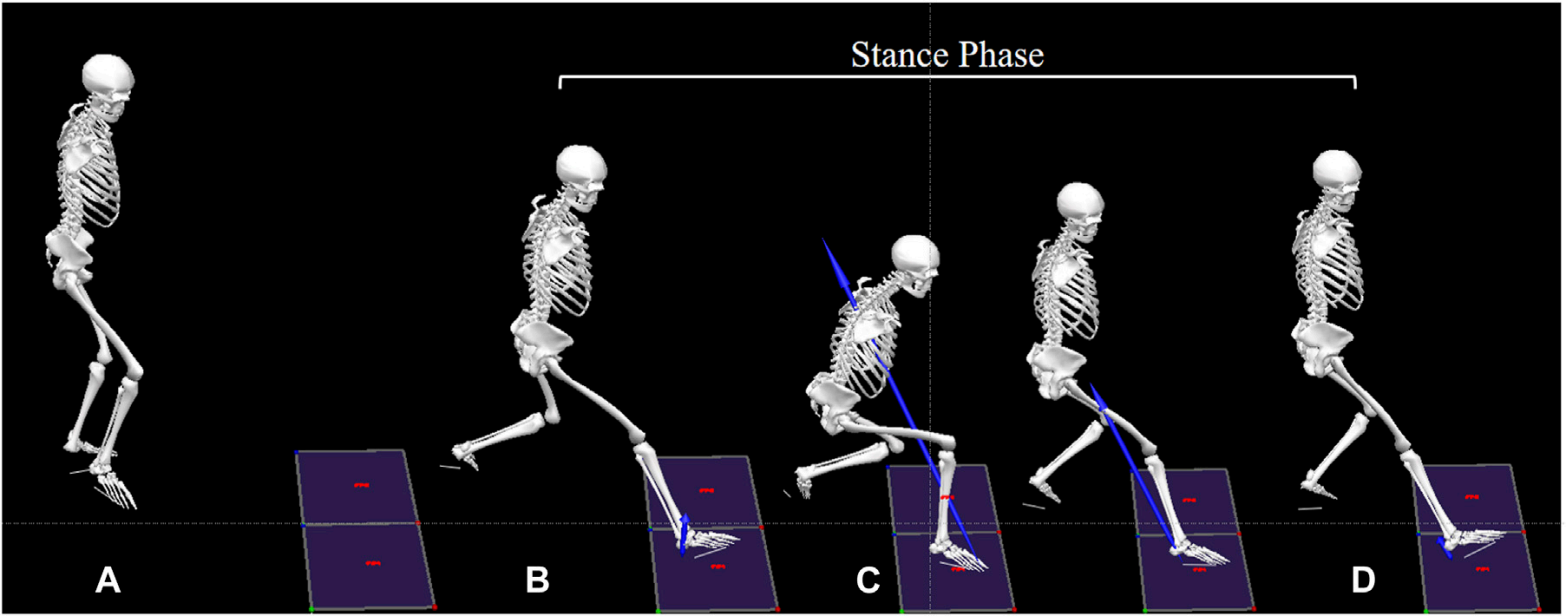
Division of lunge phases. The forehand forward lunge is used as an example to illustrate the division of lunge phases: **(A)** Preparing position: The initial standing posture before initiating the lunge; **(B)** Initial contact: The heel contacts the force plate, with vertical ground reaction force >15 N; **(C)** Moment of minimum flexion angle: The point at which the dominant knee joint achieves its minimum flexion angle during the lunge; **(D)** Lift-off: The dominant leg leaves the force plate, with vertical ground reaction force <15 N. The period from **(A, B)** represents the starting step of the lunge, **(B, C)** indicates the landing step, and **(C, D)** reflects the backing step into the preparing position. The stance phase is defined as the period from **(B, D)**.

The variables were computed during the stance phase (see Figure 3). The stance phase was defined as the period from initial contact to final lift-off of the racket-holding leg from the force plate. Contact and lift-off events were identified based on the vertical reaction force, with a cutoff threshold of 15 N (Kuntze et al., 2010). Joint stiffness was calculated as the ratio of the change in joint moment (ΔM) to the change in joint angle (Δθ), where Δθ represents the range of motion during the stance phase. The joint angle, joint moment, and GRF data of the time series during stance phase were normalized to 101 frames. All kinetic data were normalized to each participant’s body weight (BW). The mean of three valid trials for each variable was then used for analysis.

### 2.5 Statistical analysis

Prior to statistical analysis, the normality of all variables was assessed using the Shapiro–Wilk test. For variables that met the assumption of normality, paired-sample t-test were performed. If normality was not met, the Wilcoxon signed-rank test was used as a non-parametric alternative. Given the one-dimensional (1D) nature of the joint angle, joint moment, and ground reaction force (GRF) data, Statistical Parametric Mapping 1D (SPM1D) was applied to analyze data across the three planes of motion (Mei et al., 2017; Pataky et al., 2017). Paired-sample t-tests within SPM1D (http://www.spm1d.org/index.html) were use for this analysis. All statistical analyses were conducted using SPSS version 17.0 (IBM, Armonk, NY, United States), and Python version 3.12.1 (Python Software Foundation, Wilmington, DE, United States). The significance level was set at p < 0.05.

## 3 Results

**FIGURE 4:**
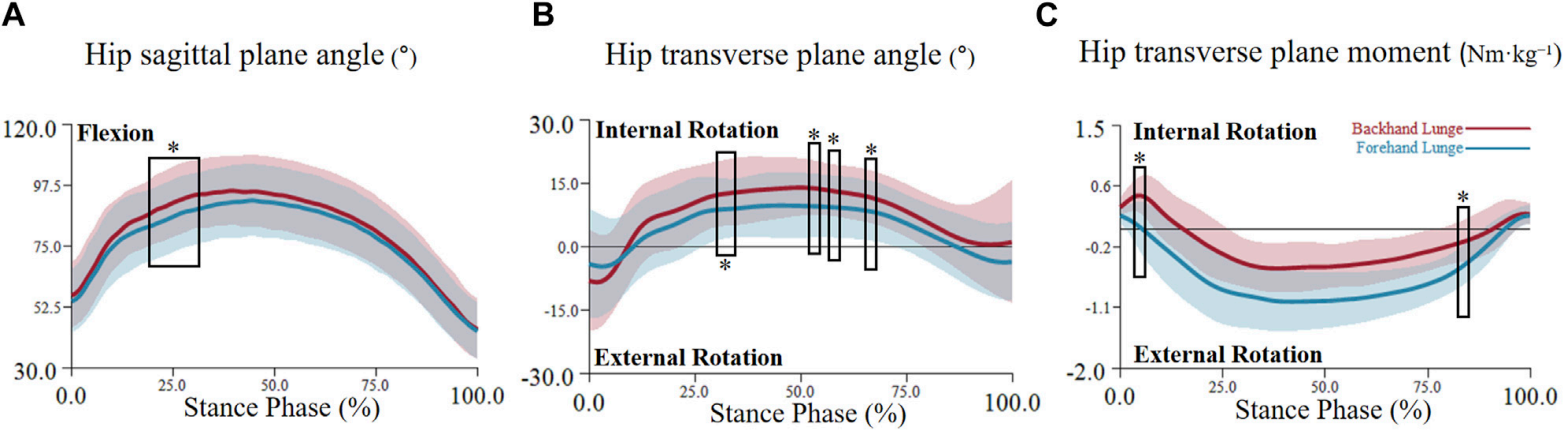
The mean (SD) value waveform of hip joint angle and moment during the stance phase (* indicates significance). **(A)** shows the hip flexion angle alterations between two lunge types during the stance phase. **(B)** shows the hip transverse plane angle alterations between two lunge types during the stance phase. **(C)** shows the hip transverse plane moment alterations between two lunge types during the stance phase.

**FIGURE 5:**
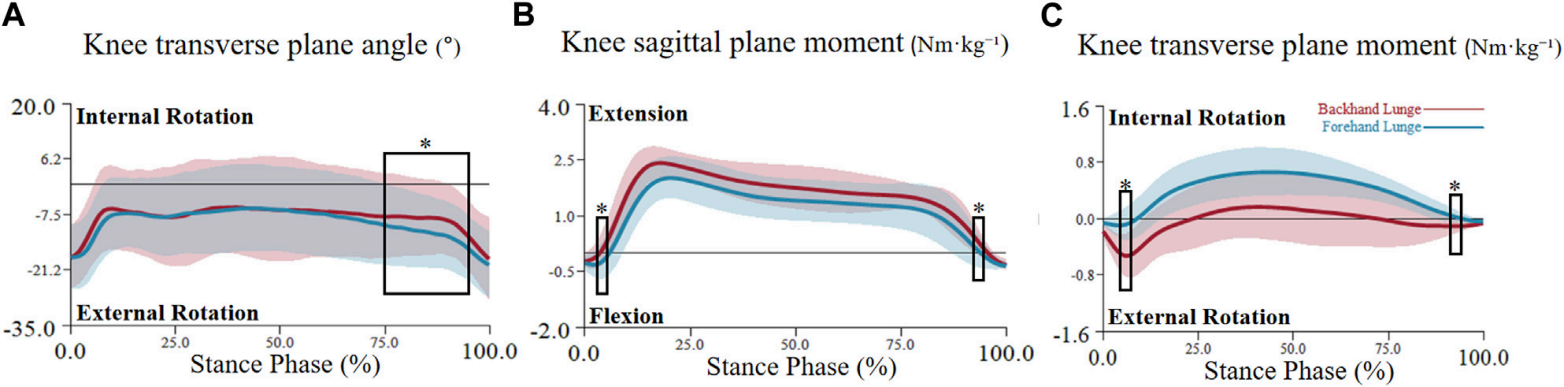
The mean (SD) value waveform of knee joint angle and moment during the stance phase (* indicates significance). **(A)** shows the knee transverse plane angle alterations between two lunge types during the stance phase. **(B)** shows the knee sagittal plane moment alterations between two lunge types during the stance phase. **(C)** shows the knee transverse plane moment alterations between two lunge types during the stance phase.

**FIGURE 6:**
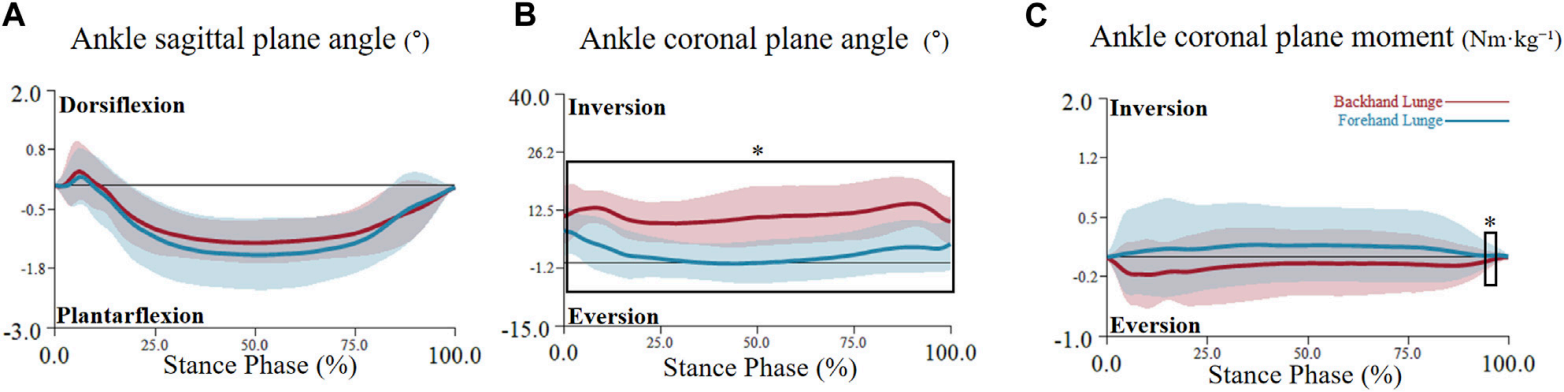
The mean (SD) value waveform of ankle joint angle and moment during the stance phase (* indicates significance). **(A)** shows the ankle sagittal plane angle alterations between two lunge types during the stance phase. **(B)** shows the ankle coronal plane angle alterations between two lunge types during the stance phase. **(C)** shows the ankle coronal plane moment alterations between two lunge types during the stance phase.

For the joint angle, the critical threshold of 3.741 was exceeded during the 20%–32% phase (p = 0.004) in the hip sagittal plane angle, indicating a significantly higher hip flexion angle in the backhand lunge. Additionally, the hip transverse plane angle surpassed the critical threshold of 4.319 during multiple phases (36%–43%, 53%–56%, 57%–61%, and 63%–66%), indicating a significantly higher hip internal rotation angle in the backhand lunge. No significant difference was found in the hip coronal plane angle (see Figure 4). For the knee, the transverse plane angle exceeded the critical threshold of 4.14 during the 75%–95% phase (p < 0.001), indicating a significantly higher knee external rotation angle in the forehand lunge. However, no significant differences were observed in the sagittal and coronal knee plane angles (see Figure 5). Regarding the ankle joint, the coronal plane angle exceeded the critical threshold of 3.527 during all phases (p < 0.001), indicating a significantly higher ankle inversion angle in the backhand lunge. No significant differences were found in the sagittal and transverse ankle plane angles (see Figure 6).

**FIGURE 7:**
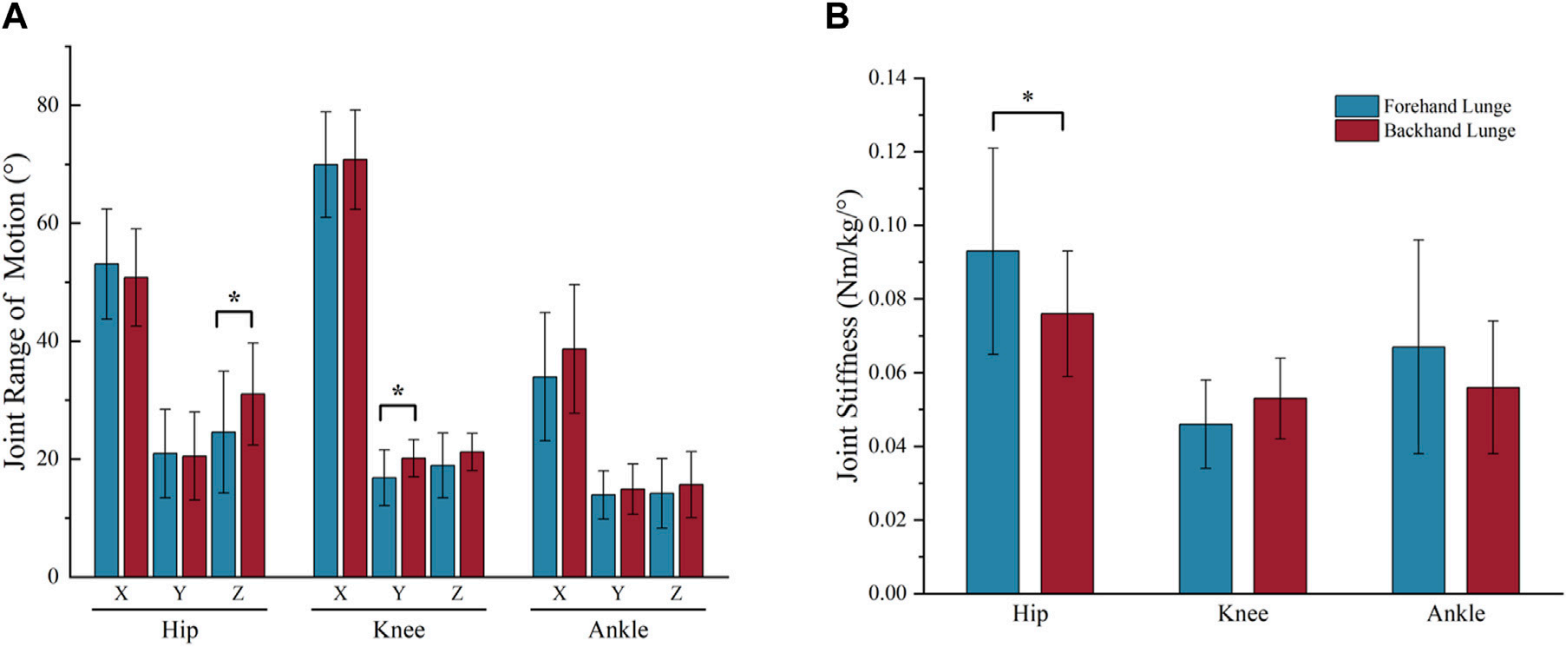
The bar chart of joint range of motion **(A)** and joint stiffness **(B)** during the stance phase (* indicates P < 0.05). In the bar chart **(A)**, X = sagittal plane, Y = coronal plane, Z = transverse plane.

For the joint range of motion (ROM), significant differences were observed in hip transverse ROM (p = 0.019) and knee coronal ROM (p = 0.023) between backhand and forehand lunges. Specifically, the knee coronal ROM was greater in the backhand lunge (20.13° ± 3.16°) compared to the forehand lunge (16.83° ± 4.75°). Similarly, hip transverse ROM was higher in the backhand lunge (31.05° ± 8.64°) than in the forehand lunge (24.58° ± 10.35°). However, no significant differences were found in the ROM of the hip sagittal and coronal planes, knee sagittal and transverse planes, or the ankle joint across all three planes (see Figure 7).

**FIGURE 8:**
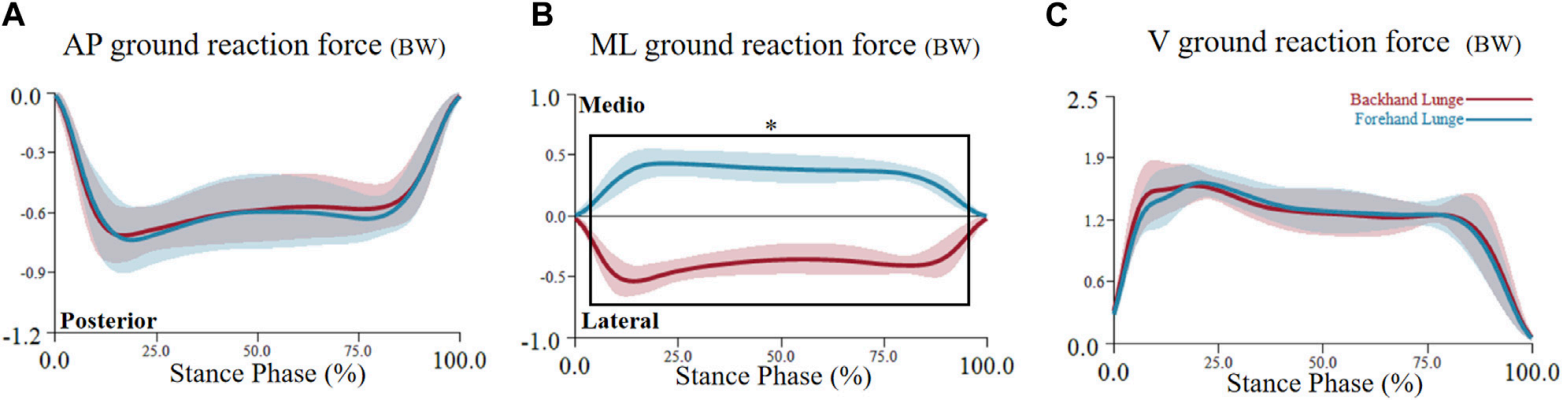
The mean (SD) value waveform of ground reaction force during the stance phase (* indicates significance). ML = medio-lateral direction, AP = anterior-posterior direction, V = vertical direction, BW = body weight. **(A)** shows the anterior-posterior ground reaction force alterations between two lunge types during the stance phase. **(B)** shows the medio-lateral ground reaction force alterations between two lunge types during the stance phase. **(C)** shows the vertical ground reaction force alterations between two lunge types during the stance phase.

For the joint ground reaction force, SPM1D analysis revealed a significant difference in the medio-lateral GRF, with one supra-threshold cluster (3%–96%) exceeding the critical threshold of 4.255 (p < 0.001). However, no significant differences were found in the antero-posterior and vertical ground reaction forces (see Figure 8).

For the joint moment, the hip transverse plane moment exceeded the critical threshold of 4.552 during the 2.8%–3.2% phase (p = 0.05) and the 80%–82% phase (p = 0.033), indicating a significantly higher hip internal rotation moment in the backhand lunge during the early phase and a higher hip external rotation moment in the forehand lunge during the late phase (see Figure 4). No significant differences were found in the hip sagittal and coronal plane moments. For the knee, the critical threshold of 4.128 was surpassed during the 4.5%–5.5% phase (p = 0.049) and the 92%–94% phase (p = 0.046), indicating a significantly higher knee flexion moment in the forehand lunge and a higher knee extension moment in the backhand lunge. Moreover, the knee transverse plane moment exceeded the critical threshold of 4.123 during the 1.5%–2.5% phase (p = 0.049) and the 92%–96% phase (p = 0.019), indicating a significantly higher knee external rotation moment in the backhand lunge. No significant differences were found in the coronal plane knee moment (see Figure 5). For the ankle, the coronal plane moment surpassed the critical threshold of 4.037 during the 97%–98% phase (p < 0.001), indicating a significantly higher ankle eversion moment in the backhand lunge. No significant differences were found in the sagittal and transverse ankle moments (see Figure 6).

For the joint stiffness, no significant differences were observed in knee and ankle stiffness. However, hip stiffness showed a significant difference (p = 0.018) between the two lunge directions. The forehand lunge exhibited significantly higher hip stiffness (0.093 ± 0.028 Nm/kg/°) compared to the backhand lunge (0.076 ± 0.017 Nm/kg/°). (see Figure 8).

## 4 Discussion

The aim of this study was to examine differences in lower limb kinematics and dynamics between forehand and backhand forward lunges in amateur female badminton players. We hypothesized that lower limb kinematics and dynamics would exhibit distinct differences between forehand and backhand lunges. The findings of this study support our hypothesis, revealing significant differences in lower limb joint angles, range of motion (ROM), joint moments, stiffness, and ground reaction forces (GRF) between the two lunge types.

Previous studies have identified smaller hip and knee flexion angles as indicators of effective lunge performance (Huang et al., 2014; Mei et al., 2017; Lee and Loh, 2019). Specifically, lunging with the knee not extending beyond the toes and with minimized hip flexion, allowing players to return to the base position more quickly (Mei et al., 2017). Although no significant differences in knee flexion angles were observed between forehand and backhand lunges in this study, the hip flexion angle was significantly larger during the 20%–32% stance phase in backhand lunges. This suggests that backhand lunges place greater demands on the lower limbs, requiring more effort to return to the base position compared to forehand lunges. Additionally, the backhand lunge exhibited greater hip internal rotation angles, a greater transverse plane ROM, and higher internal rotation moments. These findings may reflect the increased biomechanical demands associated with lunging in different forward directions, indicating that more internal hip rotation is necessary to complete backhand lunges effectively.

The lunge can be described within the framework of the stretch-shortening cycle (SSC), which is essential for developing sufficient lower-limb stiffness to store elastic energy and generate force during SSC activities (Komi, 2000; Brazier et al., 2019). Stiffness can be described as the resistance to deformation of an object in response to an applied force (Butler et al., 2003; Pruyn et al., 2014; Brazier et al., 2019). The ability to generate higher stiffness in the lower limb benefits movements (Maloney and Fletcher, 2021), like maximum-velocity running (Bret et al., 2002) or changes in direction (Serpell et al., 2014). This study found that forehand lunges exhibited higher hip stiffness compared to backhand lunges. This suggests that the hip may adapt to accommodate greater stability demands in specific directions, consistent with findings that alterations in joint stiffness accommodate varying movement directions (Dean and Kuo, 2008). During a forehand lunge, maintaining higher hip stiffness may enhance elastic energy storage and concentric force generation during push-off, offering new insights into hip function in directional lunges. Therefore, targeted training of hip-related muscle groups (especially the internal and external rotators) can improve the stepping performance of amateur female badminton players.

In addition, Huang et al. (2014) investigated trunk and knee motions, dynamic stability control, and muscle activation patterns in individuals with and without knee pain. Their study revealed that individuals with knee pain exhibited reduced knee motion in the coronal and transverse planes during the forehand forward lunge. This finding suggests that badminton players with knee pain may adopt a more conservative knee movement pattern to minimize the recurrence of pain. In the present study, we observed that, compared to the forehand lunge, the backhand lunge exhibited a greater knee coronal range of motion (ROM). This result indicates that amateur female badminton players with knee pain should exercise caution when performing backhand forward lunges during training and competition to reduce the risk of pain recurrence.

Notably, our results showed that during the backhand lunge, compared with the forehand lunge, there was a larger ankle varus angle throughout the entire stance phase. Additionally, a greater ankle valgus moment was observed only at the end of the stance phase (97%–98%). This may be because a higher ankle valgus moment is required to resist the increased varus activity during the backhand lunge. However, previous studies have shown that an increasing ankle inversion angle may elevate the risk of sustaining a lateral ankle sprain (Willems et al., 2005; Herbaut and Delannoy, 2020). Therefore, it can be inferred that amateur female badminton players face a higher risk of ankle injury during the backhand lunge. Lower limb injuries account for 52.15% of total badminton-related injuries, with ankle injuries being the most common (Marchena-Rodriguez et al., 2024). Consequently, incorporating ankle stability and preventive exercises into backhand lunge drills may help reduce the potential risk of injury.

Moreover, this study found significant differences only in the medio-lateral ground reaction forces indicating that the execution of forehand and backhand lunges is subjected to different ground reactions in the left and right directions. In addition, Nielsen et al. (2020) reported that, compared with the forehand step, the backhand lunge exhibited lower coronal moments at the hip, knee, and ankle joints. This finding suggests that backhand forward lunges may pose a lower risk of overuse injuries and discomfort. However, the present study did not observe these differences, which may be attributed to the fact that Nielsen et al.'s participants were male badminton athletes, whereas this study focused on female amateur badminton players. These findings indicate that female players exhibit different dynamic characteristics and may experience varying injury risks when performing these movements. Consequently, this study underscores the necessity for further research into the influence of skill level and gender on badminton-related injury prevention. Future research should specifically investigate gender-related differences to better understand their impact on biomechanics and injury risk.

It is worth noting that SPM1D analysis indicated this study identified numerous significant differences in the angles and moments of the three lower limb joints during the early phase (0%–10%) and the late phase (80%–100%) of the stance phase. During the early phase of lunging, the backhand lunge exhibited a larger internal rotation moment at the hip joint and a larger external rotation moment at the knee joint, as well as a smaller extension moment at the knee joint. In contrast, during the late phase, the forehand lunge demonstrated greater internal rotation moments at the hip, external rotation moments at the knee, valgus moments at the ankle, and smaller flexion moments at the knee. These findings reflect the distinct joint activity requirements and lower limb execution strategies employed during forward lunges in different directions, emphasizing the importance of both the initial and final phases of the stance. Future research on the biomechanical characteristics of these two phases may help identify key features that can inform training and injury prevention strategies for amateur female badminton players.

This study has several limitations. First, since the participants were amateur female badminton players from a sports university, the findings may not be generalizable to elite athletes. Additionally, the lunges were performed on a simulated court with a prespecified lunge distance rather than during real match conditions. Future research should aim to recruit professional badminton players and simulate competition scenarios to obtain more representative data. Moreover, further studies should explore gender differences by comparing unskilled male and female players to provide deeper insights into injury risks and movement mechanics. As mentioned earlier, differences in biomechanics between male and female players should be investigated to enhance training strategies tailored to each group. Finally, incorporating electromyography (EMG) analysis and assessing trunk biomechanics could provide a more comprehensive understanding of the key biomechanical characteristics of lunging, offering valuable guidance for injury prevention and training optimization.

## 5 Conclusion

This study investigated the biomechanical differences in the lower limbs of female amateur badminton players performing forward lunges in different directions. The results indicate that greater hip internal rotation is required for the backhand lunge. Moreover, the backhand lunge exhibited a higher ankle varus angle, which could increase the risk of ankle injury. Forehand lunges demonstrated greater hip stiffness, potentially reflecting lower limb adaptation to the demands of lunging in different directions. Additionally, numerous differences were observed in the angles and moments of the lower limb joints at the beginning and end of the lunge phase, highlighting the importance of these two phases for future research. These findings can guide the design and implementation of testing protocols and targeted training programs to enhance performance and reduce injury risk in female amateur badminton players.

## Data Availability

The raw data supporting the conclusions of this article will be made available by the authors, without undue reservation.
